# Malignant pleural mesothelioma with constrictive pericarditis as the first manifestation: A case report

**DOI:** 10.1002/ccr3.7555

**Published:** 2023-06-20

**Authors:** Cheng‐Peng He, Di‐Wei Tu, Ting‐Wei Zhang, Qian Zhang, Qiang Zhang, Di Kang, Ying‐Ying Wang, Ying‐Ying Li, Bin Zhang, Sha‐Sha Han, Hong‐Bo Li

**Affiliations:** ^1^ Department of Respiratory and Critical Care Medicine Binzhou Medical College Affiliated Hospital (First Clinical Medical College) Binzhou China; ^2^ Department of Pathology Binzhou Medical College Affiliated Hospital Binzhou China; ^3^ Department of Imaging Binzhou Medical College Affiliated Hospital Binzhou China

**Keywords:** constrictive pericarditis, malignant pleural mesothelioma, pericardium

## Abstract

Pleural mesothelioma (PM) with pericardial involvement is extremely rare. We now report a rare case of malignant PM with constrictive pericarditis as the first presentation. A 59‐year‐old male diagnosed with constrictive pericarditis underwent pericardiectomy and pericardial pathology revealed mesothelial hyperplasia. Eight months after surgery, the patient was admitted to the hospital with chest tightness and wheezing for 5 days. Computed tomography scan of the chest showed a left lung expansion insufficiency, limited bilateral pleural thickening, pericardial thickening with a small amount of pericardial effusion, and multiple enlarged lymph nodes in the mediastinum, bilateral supraclavicular fossa, bilateral cervical roots, and right axilla. The pleural malignancy should be possibly considered. Pathology after pleural puncture showed malignant PM. Pathology after left supraclavicular lymph node puncture biopsy showed metastatic malignant mesothelioma. The diagnosis of this patient was clear. Although malignant PM rarely involves the pericardial constriction, we cannot ignore the fact that malignant PM involves the pericardium. The patient has been diagnosed with constrictive pericarditis, accompanied by pleural thickening and pleural effusion. Without other pathogenic factors, pleural biopsy should be aggressively performed in patients with constrictive pericarditis to determine the cause.

## Graphical abstract

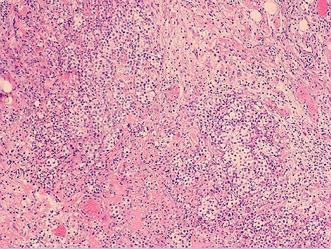

## 1 INTRODUCTION

Mesothelioma is a rare malignancy that originates in the plasma membrane and can occur in the pleura, pericardium, abdomen, and spine.^1^, ^2^, ^3^, ^4^ Pleural mesothelioma (PM) is the most common of mesotheliomas, with chest pain and dyspnea as the initial symptoms.^5^ Progressive worsening of chest pain and dyspnea as the disease progresses. However PM rarely involves the pericardium, and pericardial involvement in PM is mainly caused by direct tumor invasion. We report a case of constrictive pericarditis caused by malignant PM.

## 2 CASE PRESENTATION

A 59‐year‐old male, previously healthy and denying any other medical history, was admitted to the hospital with “chest tightness for 20 days” presenting with retrosternal chest pain that worsened upon supination. The patient had a history of smoking for more than 40 years, denied asbestos exposure, and denied family history. Computed tomography (CT) scan showed a small amount of effusion in the left pleural cavity, limited bilateral pleural thickening, and pericardial thickening. Cardiac ultrasound suggested a high probability of constrictive pericarditis without pericardial effusion. The diagnosis of constrictive pericarditis was made based on the condition and the findings of the ancillary tests, but the etiology was unknown. On the ninth day after admission, the patient was treated with pericardiectomy by the cardiac surgeon after excluding contraindications to surgery. They developed a comprehensive postoperative treatment for the patient, including anti‐infection, diuresis, adjustment of cardiac function, and other symptomatic support measures. The patient recovered well and was discharged 12 days later. Out‐of‐hospital treatment with digoxin (0.125 mg qd), furosemide (20 mg bid), and spironolactone (20 mg bid) was continued for 6 months. The cause of constrictive pericarditis is unknown, although the surgeon extracted the pericardial tissue and mediastinal lymph nodes and sent them for pathological examination. Postoperative pathology of the pericardial tissue showed fibrofatty tissue, within which mesothelial hyperplasia arranged in a striated and glandular pattern was detected. Immunohistochemistry showed: CK(+), Vimentin(−), D2‐40(+), MC(+), CR(+), CK7(+) CK20(−), Villin(−), P53 (weak +), Desmin(−), Ki‐67 (low expression). Postoperative pathology of the mediastinal lymph nodes showed reactive hyperplasia of the lymphoid tissue, within which mesothelial components were visible. Immunohistochemistry showed CK(+), D2‐40(+), MC(+), CR(+), CK7(+) CK20(−), Villin(−), TTF‐1(−), Napsin A(−), Ki‐67 (low expression). However, the surgeons at the time did not consider mesothelioma as a cause of constrictive pericarditis because of the lack of knowledge about PM‐induced constrictive pericarditis. This is why we report this case, and we would like to draw the attention of clinicians to the fact that PM is also an etiology of constrictive pericarditis.

**FIGURE 1:**
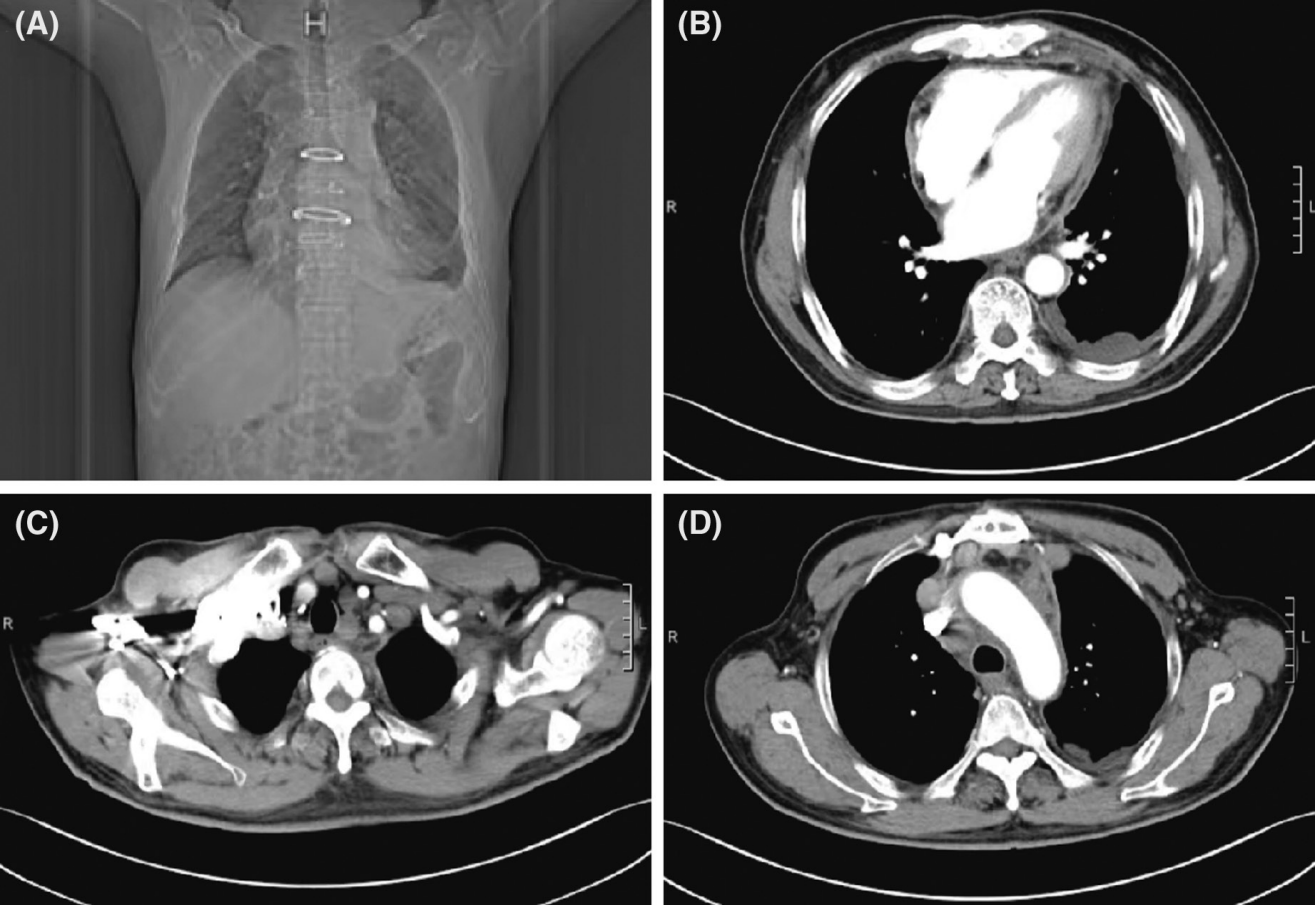
Postoperative CT (A) Sternal internal fixation. (B) Bilateral pleural thickening, left lung insufficiency, pericardial thickening combined with a small amount of pericardial effusion. (C) Enlargement of lymph nodes in the superior and inferior clavicular fossa. (D) Mediastinal lymph node enlargement.

**FIGURE 2:**
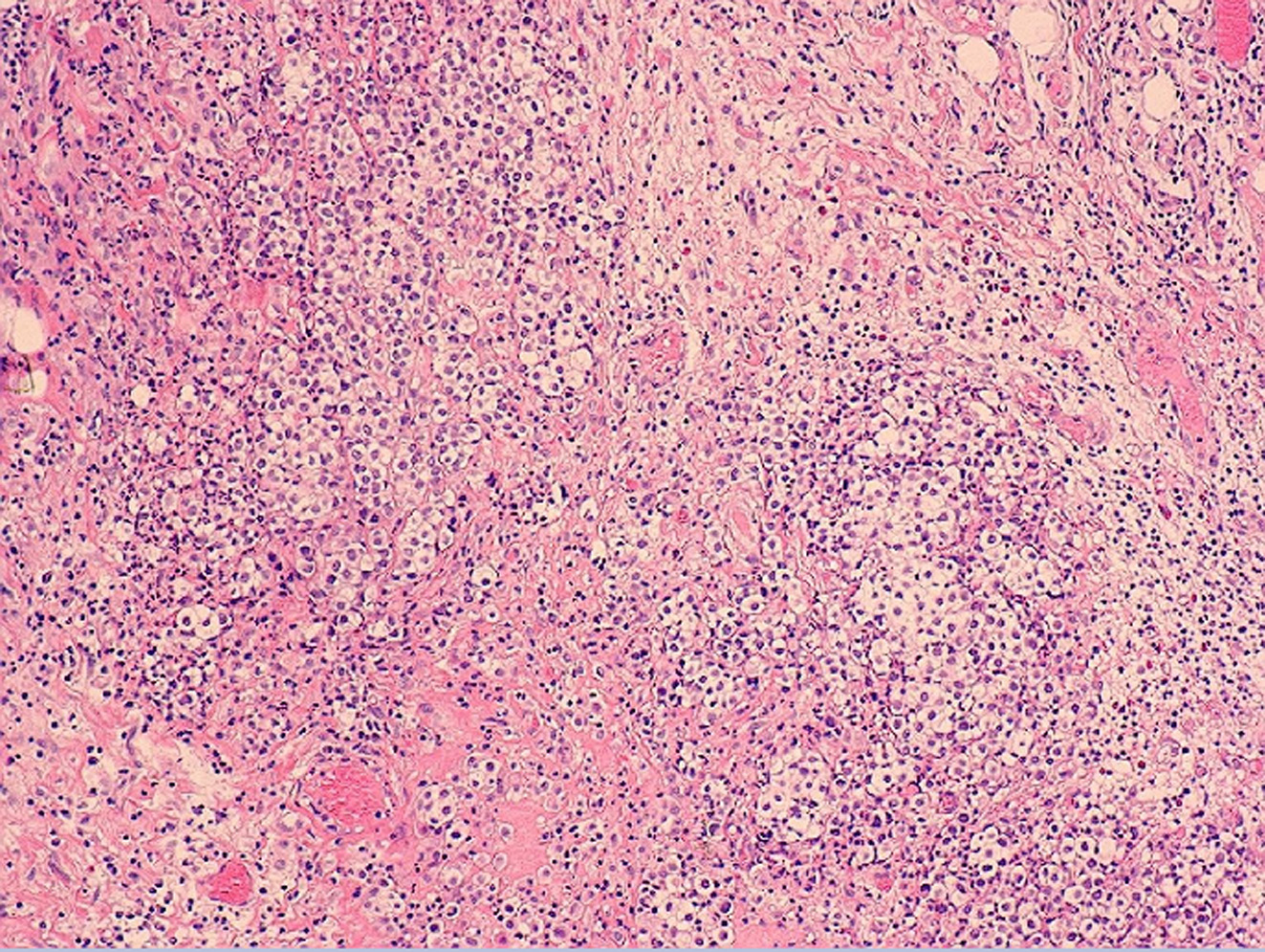
Pleural pathology: malignant mesothelioma demonstrated.

Eight months after surgery, the patient was readmitted to the hospital with chest tightness and wheezing for 5 days, presenting with left‐sided chest pain, aggravated by supine and breathing, accompanied by cough, coughing white mucous sputum, decreased appetite, and weight loss. The patient complained of poor appetite more than 20 days ago. Physical examination showed multiple lymph nodes were palpable in the anterior neck and supraclavicular region bilaterally, with hardness, poor mobility, adhesions, and tenderness on the right side; breath sounds in the left lung were weak and wet rales could be heard; head, abdominal, and neurological examinations were not significantly abnormal; examinations of both lower limbs were also unremarkable. The Chest CT (Figure 1) showed sternal fixation, full heart, thickened pericardium, and a small amount of pericardial fluid; multiple enlarged lymph nodes in the mediastinum, bilateral supraclavicular fossa, bilateral cervical roots, and right axilla; left pleural effusion and left lung insufficiency with limited pleural thickening bilaterally. Pleura pathology showed malignant mesothelioma (left mural pleura) (Figure 2). Immunohistochemistry showed CK(+), Vimentin(+), D2‐40(+), CK5/6(+), WT‐1(+), CR(+), CK7(+) TTF‐1(−), LCA(−), KI‐67(30%+). Mesothelial cells and lymphocytes were observed in the pleural effusion smear and sediment. Pathology of left supraclavicular lymph node puncture biopsy showed metastatic malignant mesothelioma. Immunohistochemistry showed D2‐40 (+), CK5/6 (+), CR (+), CK7 (+), WT‐1 (+), TTF‐1 (−), KI‐67 (30% +). The patient was ultimately diagnosed with malignant PM. Then, PP (pemetrexed 0.8gD1, carboplatin 600mgD1) regimen combined with sintilimab 200 mg treatment has been initiated. After three cycles of treatment, the patient did not continue treatment. On April 5, 2023, the patient was followed up by telephone and was in poor condition.

**FIGURE 3:**
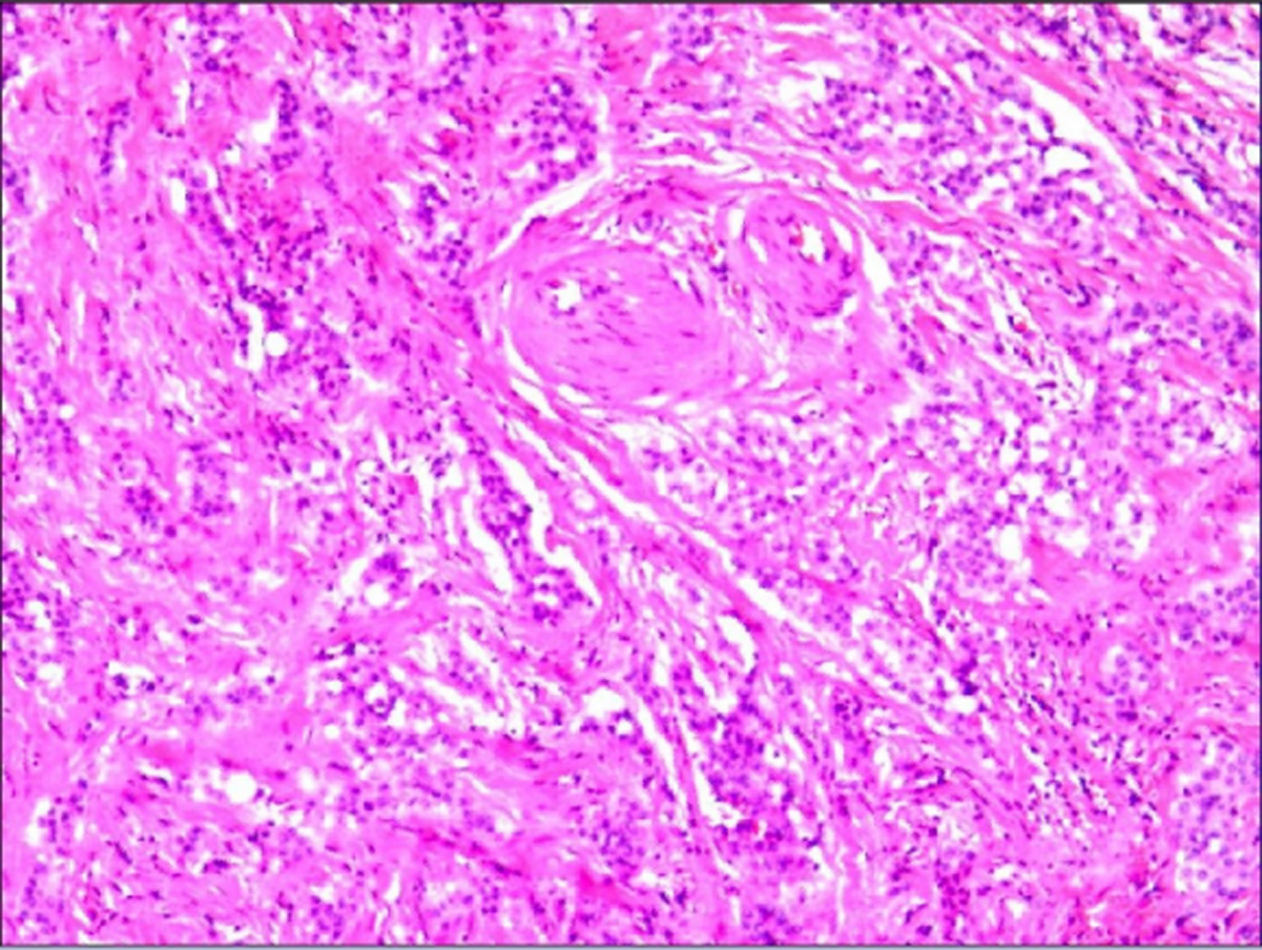
Pericardial pathology.

Reviewing the disease progression of our patient, the initial presentation was dominated by constrictive pericarditis, and after pericardiectomy, postoperative pathology (pericardium and mediastinal lymph nodes) showed mesothelial hyperplasia, although these two pathological changes may not be sufficient to diagnose mesothelioma, the patient already had pleural thickening, pleural effusion and pericardial thickening at that time, and our clinicians only performed surgical treatment for constrictive pericarditis without considering the diagnosis of PM or performing further pleural biopsy. The patient was diagnosed with malignant PM on his second admission. Reviewing his first medical history, chest CT, cardiac ultrasound, and laboratory findings from pathology, we carefully reviewed the patient's pericardial pathology biopsy from 8 months earlier, which showed mild anomalous changes in the mesothelium (Figure 3). Combining the two pathological findings, clinical manifestations and laboratory tests, we invited radiologists and pathologists in a multidisciplinary discussion of the patient's results. Finally, it was determined that the constrictive pericarditis was caused by malignant PM.

## 3 DISCUSSION

Mesothelioma is a highly malignant tumor with a high metastatic rate. In the past, asbestos exposure was the main risk factor for mesothelioma.^6^ However, with the changing nature of work, the incidence of PM is decreasing in the United States. For example, an epidemiological analysis of complete data from 1509 cases of mesothelioma registered in Norway between 2000 and 2019 showed that the incidence of PM in men decreased from 1.7 per 100,000 in 2000–2004 to 1.1 per 100,000 in 2015–2019, while the incidence in women largely stabilized at no more than 0.3 per 100,000.^7^ However, its survival rate remains low because of late detection, late diagnosis, and treatment resistance.^6^ The early clinical manifestations of MPM are either asymptomatic or insidious, with chest pain and breathing difficulties which is caused by pleural effusion being the most common. The case of Malignant PM with the early symptoms of constrictive pericarditis is infrequent.

The onset of MPM is concealed, and the symptoms are not typical. The chest CT scan shows diffuse or nodular thickening of the pleura, indicating a high likelihood of the disease, especially involving the mediastinal pleura.^8^ The preferred diagnostic method for MPM is pleural biopsy, and pleural fluid pathology is also possible, but with low sensitivity. Thoracoscopy subpleural biopsy not only obtains an accurate specimen of the lesion but also provides a comprehensive examination of the thoracic cavity. Medical thoracoscopy (MT) has a good safety profile and a high positive detection rate for MPM.^9^ Our case showed both pleural thickening and mediastinal lesions in the early stages. Finally, the patient was diagnosed with MPM through pleural biopsy.

Klima et al. retrospectively analyzed 5 of 18 cases of malignant mesothelioma in which chest surgery was performed 1–3 years prior to the diagnosis of malignant mesothelioma and the pleural pathology was diagnosed as benign lesions. Morphology of early pleural specimens and tumor specimens were then retrospectively compared in three of the five cases, and significant mesothelial cell lesions were found (the specific manifestations are crowding behavior of mesothelial cells, morphological size change and nuclear hyperchromatism of mesothelial cells), which are early warning signs of malignant mesothelioma.^10^ Weira et al. described a case of PM combined with effusive constrictive pericarditis in 2001, in which the patient initially presented with asbestos‐related bloody pleural effusion with pericardial effusion and pericardial thickening, and after 3 months of treatment, the patient died due to respiratory dysfunction.^11^ That the case is similar to the one we reported. To our knowledge, pericardial mesothelioma predisposes to constrictive pericarditis, whereas PM rarely leads to constrictive pericarditis. Therefore, the case we report is one of the few cases of malignant PM involving the pericardium (Table 1).

**TABLE 1:** Characteristics of patients with pleural mesothelioma involving pericardium.

First author/year	Gender/age (y)	Smoking or not	Exposure to asbestos or not	Primary symptoms	Clinical manifestations	Treatment	Reference
Weir/2001	Male/76	Yes	Not	No symptoms	Initially presented with pleural effusion, followed 2 years later by pericardial effusion and constrictive pericarditis	Radiotherapy and chemotherapy (not specified)	11
Jayaranagaiah/2018	Male/71	Yes	Not	Chest pain and dyspnea	Pericardial effusion	Not treated	4
Our case	Male/59	Yes	Not	Chest congestion and wheezing	Pleural effusion, pericardial effusion and constrictive pericarditis	Pemetrexed and carboplatin regimen in combination with sintilimab	

There is no particularly effective treatment method for MPM. Currently, chemical drug therapy is the primary method. The first‐line treatment of MPM is the combination chemotherapy of platinum and pemetrexed, and the addition of bevacizumab (an anti‐vascular endothelial factor drug) can significantly improve the overall survival (OS) of patients. Recently, The CheckMate 743 phase 3 study found that immunosuppressive therapy with nivolumab + ipilimumab significantly improved OS compared to platinum plus pemetrexed in 605 patients with MPM not feasible for surgical resection.^12^ Asciak et al. reviewed data from 761 patients diagnosed with MPM, and analysis found that time to pleural effusion exposure was not associated with PM survival, but that pleurodesis success in patients with MPM improved OS, suggesting that when patients are diagnosed with MPM, pleurodesis should be performed as early as possible.^13^ Additionally, thoracoscopic findings of pleural adhesions and plaques may indicate a poor prognosis for MPM.^9^

## 4 CONCLUSION

In conclusion, we believe that PM can lead to early constrictive pericarditis and is easily overlooked by our clinicians. For patients with unexplained constrictive pericarditis and pleural thickening, the possibility of malignant PM should be fully considered during the diagnosis and treatment. When a patient undergoes surgical resection for unexplained constrictive pericarditis, we should perform an early pleural biopsy at the time of surgery. When pathology reveals mesothelial cells and the diagnosis is uncertain, we can send the specimen to a specialist hospital to seek a diagnosis, and if the diagnosis is still not clear at this point, we suggest that the patient be followed up and examined regularly to exclude the possibility of malignant PM.

## AUTHOR CONTRIBUTIONS

**Chengpeng He:** Writing – original draft. **Diwei Tu:** Writing – original draft. **Tingwei Zhang:** Writing – review and editing. **Qian Zhang:** Resources; writing – review and editing. **Qiang Zhang:** Resources; writing – review and editing. **Di Kang:** Writing – review and editing. **Yingying Wang:** Writing – review and editing. **Yingying Li:** Writing – review and editing. **Bin Zhang:** Writing – review and editing. **Shasha Han:** Writing – review and editing. **Hong‐Bo Li:** Writing – review and editing.

## FUNDING INFORMATION

No financial support.

## CONFLICT OF INTEREST STATEMENT

The authors declare that they have no competing interests.

## ETHICS STATEMENT

The datasets used and/or analyzed during the current study are available from the corresponding author on reasonable request. Ethics approval and consent to participate are not applicable. Written informed consent was obtained from the patient for publication of this case report.

## CONSENT

Written informed consent was obtained from the patient to publish this report in accordance with the journal's patient consent policy.

## Data Availability

All data used and analyzed during this study are available from the corresponding author upon reasonable request.

## References

[1] Del GA , Fiori S , Gaudioso G , et al. Synchronous pleural and peritoneal malignant mesothelioma: a case report and review of literature. Int J Clin Exp Pathol. 2014;7:2484‐2489. 24966960 PMC4069956

[2] Shrestha B , Handa R , Poudel B , Hingorani R . Pericardial mesothelioma presenting as constrictive pericarditis. Cureus. 2022;14:e24270. 35602795 10.7759/cureus.24270 PMC9118672

[3] Chen F , Liu B , Yu Y , Du J , Chen D . Primary spinal malignant mesothelioma: a case report and literature review. World Neurosurg. 2018;114:211‐216. 29588242 10.1016/j.wneu.2018.03.124

[4] Jayaranagaiah A , Kariyanna PT , Chidella N , et al. Malignant pleural mesothelioma presenting with cardiac tamponade‐a rare case report and review of the literature. Clin Case Rep Rev. 2018;4:1‐4.

[5] Oviedo SP , Cagle PT . Diffuse malignant mesothelioma. Arch Pathol Lab med. 2012;136:882‐888. 22849735 10.5858/arpa.2012-0142-CR

[6] Janes SM , Alrifai D , Fennell DA . Perspectives on the treatment of malignant pleural mesothelioma. N Engl J Med. 2021;385:1207‐1218. 34551230 10.1056/NEJMra1912719

[7] Brustugun OT , Nilssen Y , Eide I . Epidemiology and outcome of peritoneal and pleural mesothelioma subtypes in Norway. A 20 year nation‐wide study. Acta Oncol. 2021;60:1250‐1256. 34313510 10.1080/0284186X.2021.1955971

[8] Scherpereel A , Opitz I , Berghmans T , et al. ERS/ESTS/EACTS/ESTRO guidelines for the management of malignant pleural mesothelioma. Eur Respir J. 2020;55:1900953. 32451346 10.1183/13993003.00953-2019

[9] Xu L , Yang Y , Wang Z , Wang X , Tong Z , Shi H . Malignant pleural mesothelioma: diagnostic value of medical thoracoscopy and long‐term prognostic analysis. BMC Pulm med. 2018;18:56. 29615010 10.1186/s12890-018-0619-3 PMC5883515

[10] Klima M , Gyorkey F . Benign pleural lesion and malignant mesothelioma. Virchows Arch A Pathol Anat Histol. 1977;376:181‐193. 145715 10.1007/BF00432395

[11] Weir NA , Gerstenhaber B . A case of pleural mesothelioma with effusive‐constrictive pericarditis. Yale J Biol Med. 2001;74:159‐163. 11501711 PMC2588717

[12] Cedres S , Felip E . 3‐year CheckMate743 outcomes: ringing in immunotherapy for the treatment of malignant pleural mesothelioma. Ann Oncol. 2022;33:457‐459. 35306158 10.1016/j.annonc.2022.03.004

[13] Asciak R , Kanellakis NI , Bibby A , et al. The association between pleural fluid exposure and survival in pleural mesothelioma. Chest. 2021;160:1925‐1933. 34119515 10.1016/j.chest.2021.05.063

